# Genetic Variant *SCL2A2* Is Associated with Risk of Cardiovascular Disease – Assessing the Individual and Cumulative Effect of 46 Type 2 Diabetes Related Genetic Variants

**DOI:** 10.1371/journal.pone.0050418

**Published:** 2012-11-21

**Authors:** Anders Borglykke, Niels Grarup, Thomas Sparsø, Allan Linneberg, Mogens Fenger, Jørgen Jeppesen, Torben Hansen, Oluf Pedersen, Torben Jørgensen

**Affiliations:** 1 Research Centre for Prevention and Health, Glostrup University Hospital, Glostrup, Denmark; 2 The Novo Nordisk Foundation Centre for Basic Metabolic Research, Faculty of Health Sciences, University of Copenhagen, Copenhagen, Denmark; 3 University Hospital of Copenhagen, Hvidovre, Denmark; 4 Faculty of Health Sciences, University of Copenhagen, Copenhagen, Denmark; 5 Department of Medicine, Glostrup University Hospital, Glostrup, Denmark; 6 Faculty of Health Sciences, University of Southern Denmark, Odense, Denmark; 7 Hagedorn Research Institute, Gentofte, Denmark; 8 Faculty of Health Sciences, University of Aarhus, Aarhus, Denmark; 9 Medical Faculty, University of Aalborg, Aalborg, Denmark; Innsbruck Medical University, Austria

## Abstract

**Aim:**

To assess the individual and combined effect of 46 type 2 diabetes related risk alleles on incidence of a composite CVD endpoint.

**Methods:**

Data from the first Danish MONICA study (N = 3523) and the Inter99 study (N = 6049) was used. Using Cox proportional hazard regression the individual effect of each risk allele on incident CVD was analyzed. Risk was presented as hazard ratios (HR) per risk allele.

**Results:**

During 80,859 person years 1441 incident cases of CVD (fatal and non-fatal) occurred in the MONICA study. In Inter99 942 incident cases were observed during 61,239 person years.

In the Danish MONICA study four gene variants were significantly associated with incident CVD independently of known diabetes status at baseline; *SLC2A2* rs11920090 (HR 1.147, 95% CI 1.027–1.283 , *P* = 0.0154), *C2CD4A* rs7172432 (1.112, 1.027–1.205 , *P* = 0.0089), *GCKR* rs780094 (1.094, 1.007–1.188 , *P* = 0.0335) and *C2CD4B* rs11071657 (1.092, 1.007–1.183 , *P* = 0.0323). The genetic score was significantly associated with increased risk of CVD (1.025, 1.010–1.041, *P* = 0.0016). In Inter99 two gene variants were associated with risk of CVD independently of diabetes; *SLC2A2* (HR 1.180, 95% CI 1.038–1.341 *P* = 0.0116) and *FTO* (0.909, 0.827–0.998, *P* = 0.0463). Analysing the two populations together we found *SLC2A2* rs11920090 (HR 1.164, 95% CI 1.070–1.267, *P* = 0.0004) meeting the Bonferroni corrected threshold for significance. *GCKR* rs780094 (1.076, 1.010–1.146, *P* = 0.0229), *C2CD4B* rs11071657 (1.067, 1.003–1.135, *P* = 0.0385) and *NOTCH2* rs10923931 (1.104 (1.001 ; 1.217 , *P* = 0.0481) were found associated with CVD without meeting the corrected threshold. The genetic score was significantly associated with increased risk of CVD (1.018, 1.006–1.031, *P* = 0.0043).

**Conclusions:**

This study showed that out of the 46 genetic variants examined only the minor risk allele of *SLC2A2* rs11920090 was significantly (*P* = 0.0005) associated with a composite endpoint of incident CVD below the threshold for statistical significance corrected for multiple testing. This potential pathway needs further exploration.

## Introduction

Although the western world have experienced a substantial decrease in the mortality of cardiovascular disease (CVD) during the last three or four decades [1] CVD is still a leading cause of morbidity and premature mortality worldwide. The decrease in mortality can be attributed to decrease in case-fatality through improved treatment but the major contribution is due to a decrease in incidence. The latter is largely the result of many years of preventive efforts targeted the classical risk factors for CVD such as smoking, elevated serum cholesterol and hypertension.

Even though the classical risk factors explain most of the risk associated with CVD there is still a part of the aetiology that lacks explanation. This is seen in cardiovascular risk prediction where established scoring schemes such as the European SCORE or the American Framingham model uses conventional risk factors to predict future risk of CVD, but still a substantial number of events occur in the proportion of the population that is not in high risk as assessed through classical risk factors.[2], [3] This has led to an increased focus on identifying new markers of risk as reflected in the long list of new biomarkers as well as exploring genetic components of CVD [2]–[4].

A still increasing number of common genetic variants associated with type 2 diabetes or associated with type 2 diabetes related phenotypes like fasting glucose, fasting insulin or 2-h glucose have been identified.[5]. Since diabetes is a well established risk factor for CVD it is very likely that some genetic variants related with diabetes also serve as metabolic risk factors for CVD. Few studies have looked at the effect of diabetes related genetic variants and their risk on cardiovascular disease and they have yielded inconsistent results [6]–[9].

The purpose of this study was to investigate whether 46 type 2 diabetes related single nucleotide polymorphisms (SNP) are associated with increased risk of incident CVD and to investigate if possible associations are influenced by diabetes status.

## Materials and Methods

### Populations

Data from the first cohort study in the Danish part of the WHO initiated MONICA study (MONICA 1) was used together with data from the Inter99 study.

MONICA 1 is a population based cohort study examined at baseline during1982-84. Participants (aged 30–60 years) were randomly selected from the western municipalities of Copenhagen County. A total of 3785 of the 4807 invited participants attended the examination. Participants with a history of CVD (self-reported doctor diagnosed) or missing information on genotypes were excluded leaving 3523 persons for analysis (table 1

**Table 1:** Baseline characteristics of the two cohorts MONICA 1 and Inter99.

	MONICA 1	Inter99
Baseline years	1982–1984	1999–2001
N	3523	6049
Age (years)	45.0±7.3	45.9±7.9
Sex (% male)	50.9%	48.1%
BMI (kg/m^2^)	24.6±3.9	26.2±4.6
Systolic blood pressure (mmHg)	123.4±16.6	129.9±17.2
Total cholesterol (mmol/l)	6.1±1.2	5.5±1.1
HDL cholesterol (mmol/l)	1.5±0.4	1.4±0.4
Triglycerides (mmol/l)	1.3±1.0	1.3±1.3
Prevalent diabetes (%)	2% a	5.3% b
Current smoking (%)	58.1%	39.1%

Continuous variables presented as mean±SD otherwise as percentage

a Self reported doctor diagnosed diabetes

b OGGT and self reported doctor diagnosed diabetes

Inter99 is a population based cohort study with baseline examination in 1999–2001. Participants (aged 30–60 years) were randomly selected from the same geographical area as MONICA 1 and similar methods for data collection were used. A total of 6784 of the 12,934 invited persons participated at baseline and after exclusion of participants with a history of self-reported doctor diagnosed CVD and missing variables 6049 subjects were included in the present analysis (table 1). A detailed description of Inter99 has been published previously.[12]

### Ethics

A written informed consent was received from all participants. The studies were approved by the local ethics committee of Copenhagen County (now Capital Region of Denmark).

### Baseline examinations

In both studies participants were invited to a thorough health examination including questionnaires, physical examinations and blood samples. Daily smoking at baseline was self reported. The data from the baseline physical examinations included body mass index (BMI), calculated as weight (kg) divided by the square of the height (m^2^). Weight was measured with participants wearing only light indoor clothes and without shoes to the nearest 0.1 kg. Height was measured to the nearest 0.5 cm. Blood pressure was measured twice in the right arm in the supine position after 5 minutes of resting. Total serum cholesterol and HDL cholesterol were measured in the morning from blood samples obtained after an overnight fasting. Details on the methodology have been published previously.[10], [12]

In MONICA 1 diabetes was self-reported doctor-diagnosed diabetes. In Inter99 diabetes was defined by an oral glucose tolerance test (OGTT) combined with self-reported information on known diabetes.[12]

### Genotyping

Forty-six SNPs previously shown to associate with type 2 diabetes, fasting plasma glucose or 2 h plasma glucose at genome-wide significance levels were genotyped by KASPar SNP Genotyping System (KBioscience, Hoddesdon, UK). Data was not used if genotyping success-rates were below 95% or if the error-rates were above 1%. Success-rates were all above 98%. Error rates estimated from re-genotyping of 120 duplicate samples were all below 0.5%. Hardy-Weinberg equilibrium was tested as an additional control and only SNPs that met the criteria (*P*>0.05) were included in the analyses.

### Ascertainment of endpoints

Cardiovascular events were defined as first ever non-fatal or fatal CVD (ICD-8: 390-448/ICD-10: I00-I79). Assessment of the cardiovascular endpoints was based on data from the Danish National Patient Registry and the Danish Register of Causes of Death.[13], [14] Individuals who had died or emigrated were identified through the Central Population Registry of Statistics Denmark. Linkage between population surveys and national registries are made possible due to a unique individual ten digit code.

Follow-up time was assigned from the date of examination in 1982-83 (MONICA 1) or 1999–2001 (Inter99) until December 31, 2010 or date of first CVD event. Data were censored for date of registered emigration from Denmark or death from causes other than CVD.

### Statistics

Participants with register documented or self-reported history of myocardial infarction or stroke at baseline or missing information on genotypes or diabetes were excluded.

The analysis of the two populations (MONICA and Inter99) was done both separately and pooled using the same statistical procedures but adjusting the pooled analysis for a potential cohort effect. Cox proportional hazard regression with age as the underlying time-scale was used to analyse the effect of each SNP on the risk of incident CVD. The proportional hazard assumption of the Cox regression model was tested and met using Schoenfeld residuals.

Risk was presented as hazard ratios (HR) per risk allele. Analysis was performed in several models assessing the crude association, adjusting for sex and finally adjusting for diabetes status at baseline in order to examine if any possible association was mediated through diabetes status. In further analyses we tested whether BMI, blood pressure and fasting serum values of total cholesterol and HDL cholesterol had any mediating effect.

A genetic score summing the number of risk alleles of the 46 genetic variants was created using the same procedures as previous studies.[6], [8] An unweighted approach was chosen since the current literature was not able to provide estimates on the effect of the genetic variants on CVD

All analyses were conducted using the statistical software program SAS version 9.2 (SAS Institute Inc, Cary, NC) with a statistical significance level of 5%.

## Results

During 80,859 person years of follow-up (mean 23 years) 1441 (41%) of the 3523 participants in MONICA 1 experienced a CVD event. In Inter99 942 events (16%) in 6049 participants were registered during 61,239 years of follow-up (mean 10 years).

Table 2 shows the association of the 46 type 2 diabetes related genetic variants with incident CVD in MONICA 1 and Inter99 with and without the adjustment for diabetes status.

**Table 2:** Associations of diabetes susceptibility variants with risk of incident CVD in MONICA 1 and Inter99.

			MONICA 1					Inter99				
Gene	SNP^a^	Risk allele^b^/other	RAF^c^ (%)	Model 1^d^ HR (95% CI)	*P*	Model 2^e^ HR (95% CI)	*P*	RAF^c^ (%)	Model 1^d^ HR (95% CI)	*P*	Model 2^e^ HR (95% CI)	*P*
Variants with an effect on β-cell function^f^												
*ADRA2A*	rs10885122	G/T	89.3	1.113 (0.973 ; 1.274)	0.1188	1.105 (0.965 ; 1.266)	0.1488	88.5	0.915 (0.794 ; 1.055)	0.2209	0.918 (0.797 ; 1.059)	0.2404
*CENTD2*	rs1552224	T/G	82.0	1.011 (0.910 ; 1.123)	0.8450	1.009 (0.908 ; 1.121)	0.8671	82.3	0.927 (0.823 ; 1.045)	0.2140	0.932 (0.828 ; 1.050)	0.2478
*DGKB*	rs2191349	G/T	47.7	1.072 (0.991 ; 1.160)	0.0840	1.071 (0.989 ; 1.159)	0.0902	50.7	0.966 (0.882 ; 1.059)	0.4649	0.967 (0.883 ; 1.059)	0.4704
*KCNQ1*	rs231362	T/C	50.3	0.963 (0.890 ; 1.042)	0.3513	0.972 (0.898 ; 1.052)	0.4787	50.5	1.035 (0.944 ; 1.135)	0.4657	1.043 (0.951 ; 1.144)	0.3705
*PROX1*	rs340874	C/T	54.6	0.995 (0.919 ; 1.077)	0.8964	0.992 (0.916 ; 1.074)	0.8469	53.8	1.045 (0.952 ; 1.146)	0.3552	1.022 (0.932 ; 1.122)	0.6385
*G6PC2*	rs560887	G/A	68.6	1.011 (0.928 ; 1.101)	0.8009	1.005 (0.922 ; 1.094)	0.9150	69.9	1.047 (0.946 ; 1.158)	0.3794	1.050 (0.949 ; 1.163)	0.3423
*GLIS3*	rs7034200	C/A	52.6	0.957 (0.884 ; 1.036)	0.2808	0.960 (0.887 ; 1.039)	0.3131	47.9	1.022 (0.930 ; 1.124)	0.6466	1.020 (0.927 ; 1.121)	0.6887
*MADD*	rs7944584	A/T	74.8	0.949 (0.866 ; 1.040)	0.2663	0.951 (0.868 ; 1.042)	0.2830	75.5	1.075 (0.964 ; 1.200)	0.1943	1.065 (0.954 ; 1.188)	0.2641
*CDKN2A*	rs10811661	T/C	84.3	1.066 (0.956 ; 1.189)	0.2483	1.066 (0.956 ; 1.189)	0.2516	83.5	0.979 (0.865 ; 1.108)	0.1132	0.964 (0.852 ; 1.092)	0.5683
*MTNR1B*	rs10830963	G/C	27.0	1.019 (0.933 ; 1.113)	0.6774	1.010 (0.924 ; 1.104)	0.8259	27.3	0.971 (0.876 ; 1.076)	0.5726	0.971 (0.876 ; 1.077)	0.5808
*HHEX*	rs1111875	G/A or C/T	59.0	0.987 (0.912 ; 1.068)	0.7465	0.986 (0.911 ; 1.068)	0.7337	58.8	0.938 (0.856 ; 1.028)	0.1718	0.936 (0.853 ; 1.027)	0.1601
*CDC123*	rs12779790	G/A	19.1	1.019 (0.922 ; 1.127)	0.7088	1.032 (0.933 ; 1.141)	0.5411	19.7	0.922 (0.818 ; 1.038	0.1786	0.913 (0.810 ; 1.029)	0.1354
*SLC30A8*	rs13266634	C/T	68.5	1.001 (0.920 ; 1.089)	0.9819	1.007 (0.926 ; 1.096)	0.8652	67.8	0.987 (0.897 ; 1.087)	0.7963	1.003 (0.911 ; 1.104)	0.9559
*IGF2BP2*	rs4402960	T/G	30.1	1.000 (0.917 ; 1.090)	0.9967	0.996 (0.913 ; 1.086)	0.9283	30.5	1.031 (0.933 ; 1.139)	0.5550	1.027 (0.929 ; 1.135)	0.6060
*THADA*	rs7578597	T/C	89.6	0.999 (0.878 ; 1.137)	0.9896	1.003 (0.881 ; 1.142)	0.9627	89.6	0.934 (0.803 ; 1.086)	0.3729	0.931 (0.802 ; 1.082)	0.3541
*TCF7L2*	rs7903146	T/C	28.1	1.066 (0.977 ; 1.163)	0.1490	1.071 (0.982 ; 1.169)	0.1214	27.0	1.032 (0.932 ; 1.143)	0.5453	1.014 (0.915 ; 1.123)	0.7911
*TSPAN8*	rs7961581	C/T	25.8	1.012 (0.923 ; 1.109)	0.8072	1.015 (0.925 ; 1.113)	0.7543	27.0	1.045 (0.942 ; 1.159)	0.4031	1.020 (0.920 ; 1.131)	0.7085
*JAZF1*	rs864745	A/G or T/C	50.3	1.054 (0.973 ; 1.141)	0.1942	1.059 (0.978 ; 1.147)	0.1580	51.7	1.007 (0.918 ; 1.106)	0.8765	1.013 (0.923 ; 1.112)	0.7881
*CDKAL1*	rs7756992	G/A	29.0	1.086 (0.997 ; 1.183)	0.0583	1.082 (0.994 ; 1.179)	0.0697	28.4	1.006 (0.909 ; 1.114)	0.9034	0.999 (0.902 ; 1.106)	0.9857
*GIPR*	rs10423928	A/T	23.2	0.991 (0.903 ; 1.088)	0.8513	0.992 (0.904 ; 1.088)	0.8617	22.4	1.024 (0.917 ; 1.143)	0.6753	1.002 (0.898 ; 1.118)	0.9750
*C2CD4B*	rs11071657	A/G	61.6	**1.088 (1.004 ; 1.179)**	0.0404	**1.092 (1.007 ; 1.183)**	0.0323	61.6	1.040 (0.944 ; 1.145)	0.4256	1.034 (0.938 ; 1.139)	0.5023
*GCK*	rs4607517	A/G	15.5	0.974 (0.872 ; 1.088)	0.6431	0.960 (0.859 ; 1.073)	0.4702	15.6	1.040 (0.916 ; 1.182)	0.5455	1.022 (0.900 ; 1.161)	0.7346
*HNF1A*	rs7957197	T/A	79.3	1.002 (0.910 ; 1.103)	0.9740	1.002 (0.910 ; 1.104)	0.9635	79.8	1.036 (0.923 ; 1.163)	0.5515	1.021 (0.909 ; 1.147)	0.7221
*HNF1B*	rs7501939	T/C	59.7	0.928 (0.858 ; 1.005)	0.0657	0.928 (0.857 ; 1.004)	0.0639	60.0	1.061 (0.964 ; 1.168)	0.2269	1.068 (0.970 ; 1.175)	0.1820
*C2CD4A*	rs7172432	A/G	56.2	**1.110 (1.025 ; 1.202)**	0.0103	**1.112 (1.027 ; 1.205)**	0.0089	55.8	0.947 (0.862 ; 1.040)	0.2534	0.951 (0.865 ; 1.044)	0.2901
Variants with an effect on insulin sensitivity												
*GCKR*	rs780094	G/A	64.4	**1.090 (1.004 ; 1.184)**	0.0398	**1.094 (1.007 ; 1.188)**	0.0335	65.5	1.048 (0.950 ; 1.155)	0.3525	1.048 (0.950 ; 1.156)	0.3479
*PPARG*	rs1801282	C/G	86.7	0.976 (0.869 ; 1.095)	0.6761	0.964 (0.859 ; 1.083)	0.5382	86.3	1.098 (0.958 ; 1.258)	0.1807	1.105 (0.964 ; 1.267)	0.1504
*ADAMTS9*	rs4607103	C/T	77.9	1.035 (0.939 ; 1.141)	0.4929	1.030 (0.934 ; 1.135)	0.5566	77.2	0.921 (0.827 ; 1.025)	0.1326	0.923 (0.829 ; 1.027)	0.1416
*IGF1*	rs35767	C/T	84.7	1.088 (0.971 ; 1.218)	0.1453	1.082 (0.967 ; 1.212)	0.1706	85.3	1.039 (0.910 ; 1.187)	0.5694	1.029 (0.900 ; 1.175)	0.6789
Variant with an effect on adiposity												
*FTO*	rs8050136	A/C	40.2	1.022 (0.942 ; 1.108)	0.6036	1.024 (0.944 ; 1.111)	0.5664	41.0	**0.909 (0.827 ; 0.998)**	0.0461	**0.909 (0.827 ; 0.998)**	0.0463
Variants with unknown physiology												
*FADS1*	rs174550	A/G	66.2	1.016 (0.932 ; 1.108)	0.7135	1.012 (0.928 ; 1.103)	0.7948	66.5	0.977 (0.886 ; 1.076)	0.6327	0.976 (0.886 ; 1.075)	0.6228
*CRY2*	rs11605924	A/C	48.7	1.037 (0.957 ; 1.124)	0.3755	1.036 (0.956 ; 1.124)	0.3870	48.9	0.980 (0.893 ; 1.077)	0.6794	0.983 (0.895 ; 1.079)	0.7207
*ZFAND6*	rs11634397	G/A	67.4	1.006 (0.924 ; 1.095)	0.8948	1.021 (0.937 ; 1.112)	0.6398	66.8	1.048 (0.948 ; 1.159)	0.3550	1.045 (0.945 ; 1.155)	0.3907
*ADCY5*	rs11708067	A/G	74.5	0.990 (0.906 ; 1.083)	0.8334	0.991 (0.906 ; 1.084)	0.8485	75.4	1.055 (0.946 ; 1.177)	0.3375	1.035 (0.928 ; 1.155)	0.5335
*SLC2A2*	rs11920090	A/T	13.7	**1.148 (1.028 ; 1.283)**	0.0146	**1.147 (1.027 ; 1.283)**	0.0154	13.6	**1.153 (1.014 ; 1.310)**	0.0296	**1.180 (1.038 ; 1.341)**	0.0116
*CHCHD9*	rs13292136	C/T	91.7	1.018 (0.881 ; 1.178)	0.8047	1.022 (0.883 ; 1.182)	0.7705	92.4	0.970 (0.816 ; 1.152)	0.7276	0.968 (0.815 ; 1.151)	0.7141
*HMGA2*	rs1531343	C/G	8.46	1.016 (0.885 ; 1.167)	0.8190	0.995 (0.865 ; 1.143)	0.9390	7.9	0.980 (0.827 ; 1.160)	0.8135	0.947 (0.799 ; 1.122)	0.5276
*BCL11A*	rs243021	T/C	49.3	0.974 (0.901 ; 1.054)	0.5186	0.979 (0.904 ; 1.059)	0.5914	49.3	1.070 (0.975 ; 1.173)	0.1527	1.061 (0.967 ; 1.164)	0.2109
*ZBED3*	rs4457053	G/A	29.1	0.978 (0.896 ; 1.069)	0.6275	0.972 (0.889 ; 1.061)	0.5241	28.2	0.973 (0.878 ; 1.078)	0.5955	0.969 (0.874 ; 1.074)	0.5425
*DUSP9*	rs5945326	A/G	75.8	0.981 (0.911 ; 1.057)	0.6146	0.984 (0.914 ; 1.060)	0.6750	75.6	0.999 (0.915 ; 1.090)	0.9744	0.988 (0.905 ; 1.079)	0.7904
*PRC1*	rs8042680	A/C	30.0	1.037 (0.952 ; 1.130)	0.4022	1.033 (0.948 ; 1.126)	0.4596	29.7	1.014 (0.917 ; 1.122)	0.7812	1.006 (0.910 ; 1.112)	0.9120
*TP53INP1*	rs896854	A/G	54.5	1.058 (0.975 ; 1.147)	0.1752	1.059 (0.977 ; 1.149)	0.1644	54.1	0.989 (0.902 ; 1.085)	0.8173	1.002 (0.914 ; 1.099)	0.9631
*KLF14*	rs972283	G/A	50.3	**1.084 (1.002 ; 1.174)**	0.0452	1.080 (0.998 ; 1.169)	0.0562	50.7	1.011 (0.920 ; 1.110)	0.8237	1.014 (0.923 ; 1.114)	0.7726
*NOTCH2*	rs10923931	T/G	10.2	1.092 (0.963 ; 1.239)	0.1698	1.109 (0.977 ; 1.258)	0.1097	9.5	1.102 (0.943 ; 1.287)	0.2221	1.097 (0.939 ; 1.281)	0.2425
*KCNQ1*	rs2237895	C/A	41.5	1.056 (0.975 ; 1.144)	0.1787	1.062 (0.981 ; 1.150)	0.1374	41.3	0.986 (0.896 ; 1.085)	0.7724	0.965 (0.877 ; 1.062)	0.4683
*VPS13C*	rs17271305	G/A	39.9	0.964 (0.890 ; 1.043)	0.3611	0.966 (0.891 ; 1.046)	0.3902	40.1	1.017 (0.926 ; 1.118)	0.7225	1.024 (0.931 ; 1.125)	0.6257
Gene score				**1.025 (1.009 ; 1.041)**	0.0016	**1.025 (1.010 ; 1.041)**	0.0016		1.012 (0.990 ; 1.034)	0.2801	1.008 (0.987 ; 1.030)	0.4657

a SNP, single nucleotide polymorphism

b According to type 2 diabetes risk increasing allele in original reports

c RAF, risk allele frequency

d Model 1 adjusted for sex and age. HR is per risk increasing allele

e Model 2 adjusted for sex, age and prevalent diabetes. HR is per risk increasing allele

f According to [5]

In the MONICA 1 study four genetic variants were significantly associated with incident CVD independently of baseline diabetes status; minor risk allele of *SLC2A2* rs11920090 (HR 1.147, 95% CI 1.027–1.283 , *P* = 0.0154), major risk allele of *C2CD4A* rs7172432 (1.112, 1.027–1.205 , *P* = 0.0089), major risk allele of *GCKR* rs780094 (1.094, 1.007–1.188 , *P* = 0.0335) and major risk allele of *C2CD4B* rs11071657 (1.092, 1.007–1.183 , *P* = 0.0323) (table 2. The association became, however, statistically insignificant when adjusted for prevalent diabetes.

A genetic score summing the number of risk alleles of the 46 genetic variants was significantly associated with increased risk of CVD (1.025, 1.010–1.041 , *P* = 0.0016) per diabetes risk increasing allele even after adjustment for diabetic status. Further analysis of the ability of the genetic score to predict CVD risk in MONICA 1 showed that the c-statistics (AUC) was not significantly improved when adding the genetic score to a model including age and sex (AUC = 0.703 vs. 0.707 *P* for difference 0.1223) or to a model including age, sex, smoking, blood pressure and total cholesterol (0.725 vs. 0.729 *P* for difference 0.0665).

In Inter99 we found two genetic variants statistically associated with incident CVD; minor risk allele of *SLC2A2* rs11920090 (HR 1.180, 95% CI 1.038–1.341 *P* = 0.0116) and the minor risk allele of *FTO* rs8050136 (0.909, 0.827–0.998 ,*P* = 0.0463) (table

The results from both populations persisted after further adjusting for possible mediating factors: BMI, systolic and diastolic blood pressure and fasting serum concentrations of total cholesterol and HDL cholesterol (results not shown).

When analysing the two populations as one we find the same statistically significant association regarding minor risk allele of *SLC2A2* rs11920090 (HR 1.164, 95% CI 1.070–1.267 , *P* = 0.0004) (table

**Table 3:** Associations of diabetes susceptibility variants with risk of incident CVD.

			Pooled analysis			
Gene	SNP^a^	Risk allele^b^/other	Model 1^c^ HR (95% CI)	*P*	Model 2^d^ HR (95% CI)	*P*
Variants with an effect on β-cell function						
*ADRA2A*	rs10885122	G/T	1.021 (0.926 ; 1.126)	0.6795	1.017 (0.922 ; 1.122)	0.7320
*CENTD2*	rs1552224	T/G	0.971 (0.898 ; 1.051)	0.4680	0.973 (0.899 ; 1.052)	0.4887
*DGKB*	rs2191349	G/T	1.026 (0.967 ; 1.090)	0.3936	1.026 (0.966 ; 1.089)	0.4069
*KCNQ1*	rs231362	T/C	0.993 (0.936 ; 1.055)	0.8307	1.002 (0.944 ; 1.064)	0.9485
*PROX1*	rs340874	C/T	1.018 (0.958 ; 1.081)	0.5714	1.007 (0.948 ; 1.070)	0.8114
*G6PC2*	rs560887	G/A	1.025 (0.961 ; 1.095)	0.4524	1.023 (0.959 ; 1.092)	0.4772
*GLIS3*	rs7034200	C/A	0.982 (0.924 ; 1.044)	0.5672	0.983 (0.925 ; 1.045)	0.5895
*MADD*	rs7944584	A/T	1.000 (0.932 ; 1.073)	0.9968	0.997 (0.930 ; 1.070)	0.9396
*CDKN2A*	rs10811661	T/C	1.027 (0.946 ; 1.115)	0.5193	1.021 (0.941 ; 1.109)	0.2577
*MTNR1B*	rs10830963	G/C	0.997 (0.932 ; 1.066)	0.9301	0.992 (0.927 ; 1.061)	0.8087
*HHEX*	rs1111875	G/A or C/T	0.968 (0.912 ; 1.028)	0.2945	0.967 (0.911 ; 1.027)	0.2768
*CDC123*	rs12779790	G/A	0.978 (0.906 ; 1.056)	0.5677	0.982 (0.909 ; 1.061)	0.6455
*SLC30A8*	rs13266634	C/T	0.994 (0.933 ; 1.059)	0.8539	1.004 (0.942 ; 1.069)	0.9124
*IGF2BP2*	rs4402960	T/G	1.013 (0.949 ; 1.081)	0.7027	1.009 (0.945 ; 1.078)	0.7864
*THADA*	rs7578597	T/C	0.971 (0.880 ; 1.071)	0.5573	0.972 (0.881 ; 1.073	0.5761
*TCF7L2*	rs7903146	T/C	1.050 (0.983 ; 1.122)	0.1454	1.046 (0.979 ; 1.117)	0.1871
*TSPAN8*	rs7961581	C/T	1.025 (0.957 ; 1.098)	0.4831	1.016 (0.948 ; 1.088)	0.6525
*JAZF1*	rs864745	A/G or T/C	1.034 (0.973 ; 1.098)	0.2827	1.039 (0.978 ; 1.104)	0.2130
*CDKAL1*	rs7756992	G/A	1.051 (0.985 ; 1.123)	0.1329	1.046 (0.980 ; 1.117)	0.1754
*GIPR*	rs10423928	A/T	1.003 (0.935 ; 1.077)	0.9261	0.996 (0.928 ; 1.069)	0.9151
*C2CD4B*	rs11071657	A/G	**1.067 (1.003 ; 1.315)**	0.0384	**1.067 (1.003 ; 1.135)**	0.0385
*GCK*	rs4607517	A/G	0.998 (0.918 ; 1.085)	0.9666	0.982 (0.903 ; 1.068)	0.6670
*HNF1A*	rs7957197	T/A	1.015 (0.943 ; 1.093)	0.6891	1.011 (0.938 ; 1.088)	0.7801
*HNF1B*	rs7501939	T/C	0.980 (0.922 ; 1.042)	0.5234	0.982 (0.924 ; 1.044)	0.5619
*C2CD4A*	rs7172432	A/G	1.039 (0.977 ; 1.104)	0.2208	1.041 (0.980 ; 1.106)	0.1936
Variants with an effect on insulin sensitivity						
*GCKR*	rs780094	G/A	**1.073 (1.008 ; 1.143)**	0.0277	**1.076 (1.010 ; 1.146)**	0.0229
*PPARG*	rs1801282	C/G	1.028 (0.941 ; 1.122)	0.5457	1.023 (0.937 ; 1.118)	0.6070
*ADAMTS9*	rs4607103	C/T	0.983 (0.915 ; 1.057)	0.6416	0.981 (0.913 ; 1.055)	0.6088
*IGF1*	rs35767	C/T	1.067 (0.979 ; 1.163)	0.1421	1.059 (0.972 ; 1.155)	0.1925
Variant with an effect on adiposity						
*FTO*	rs8050136	A/C	0.973 (0.915 ; 1.034)	0.3746	0.974 (0.916 ; 1.036)	0.4100
Variants with unknown physiology						
*FADS1*	rs174550	A/G	0.999 (0.937 ; 1.066)	0.9809	0.996 (0.934 ; 1.062)	0.9038
*CRY2*	rs11605924	A/C	1.015 (0.954 ; 1.078)	0.6432	1.015 (0.955 ; 1.079)	0.6357
*ZFAND6*	rs11634397	G/A	1.025 (0.961 ; 1.094)	0.4512	1.033 (0.968 ; 1.103)	0.3254
*ADCY5*	rs11708067	A/G	1.012 (0.944 ; 1.084)	0.7356	1.005 (0.938 ; 1.077)	0.8878
*SLC2A2*	rs11920090	A/T	**1.153 (1.061 ; 1.254)**	0.0009	**1.164 (1.070 ; 1.267)**	0.0004
*CHCHD9*	rs13292136	C/T	0.997 (0.892 ; 1.114)	0.9533	0.998 (0.893 ; 1.116)	0.9731
*HMGA2*	rs1531343	C/G	1.004 (0.902 ; 1.117)	0.9455	0.978 (0.879 ; 1.090)	0.6913
*BCL11A*	rs243021	T/C	1.011 (0.952 ; 1.073)	0.7198	1.010 (0.951 ; 1.073)	0.7432
*ZBED3*	rs4457053	G/A	0.975 (0.912 ; 1.042)	0.4596	0.969 (0.907 ; 1.037)	0.3639
*DUSP9*	rs5945326	A/G	0.989 (0.935 ; 1.047)	0.7146	0.988 (0.933 ; 1.045)	0.6683
*PRC1*	rs8042680	A/C	1.029 (0.964 ; 1.099)	0.3831	1.024 (0.959 ; 1.093)	0.4790
*TP53INP1*	rs896854	A/G	1.027 (0.967 ; 1.092)	0.3865	1.033 (0.972 ; 1.098)	0.2908
*KLF14*	rs972283	G/A	1.052 (0.991 ; 1.118)	0.0998	1.051 (0.989 ; 1.117)	0.1076
*NOTCH2*	rs10923931	T/G	1.096 (0.994 ; 1.209)	0.0667	**1.104 (1.001 ; 1.217)**	**0.0481**
*KCNQ1*	rs2237895	C/A	1.025 (0.964 ; 1.089)	0.4336	1.020 (0.959 ; 1.084)	0.5358
*VPS13C*	rs17271305	G/A	0.988 (0.930 ; 1.050)	0.6928	0.992 (0.933 ; 1.054)	0.7960
Gene score			**1.020 (1.007 ; 1.033)**	0.0018	**1.018 (1.006 ; 1.031)**	0.0043

a SNP, single nucleotide polymorphism

b According to type 2 diabetes risk increasing allele in original reports

c Model 1 adjusted for sex, age and cohort. HR is per risk increasing allele

d Model 2 adjusted for sex, age, cohort and prevalent diabetes. HR is per risk increasing allele

## Discussion

The analyses showed that out of the 46 genetic variants examined only four type 2 diabetes genetic risk variants in *SLC2A2, C2CD4A, GCKR* and *C2CD4B* were associated with incident CVD in MONICA 1. In Inter99 only the *SLC2A2* and the *FTO* variant was significantly associated with CVD. Analyses of the two populations combined showed significant associations between *SLC2A2, GCKR* and *C2CD4B* respectively and CVD, but only *SLC2A2* met the threshold after correction for multiple testing. When assessing the combined effect of the 46 genetic variants the gene score was significantly associated with CVD in MONICA 1 and in the pooled analysis but not in Inter99 and had limited, if any, effect on CVD risk assessment. .

In a previous publication by Pfister and colleagues[6] they assessed the impact of 38 type 2 diabetes genetic variants on incident coronary heart disease (CHD) in a study sample comprising 20,467 participants of the European Prospective Investigation into Cancer and Nutrition (EPIC) Norfolk Study who had been free of CHD at baseline and who had a mean follow-up of 10.7 years. Only a single genetic variant (*CDKN2A/B* rs564398) associated significantly with CHD after adjustment for diabetes. In the present paper we examined the major risk allele of *CDKN2A* rs10811661 for association to CVD events but failed to demonstrate any relationship. The SNP we examined (rs10811661) is not in LD (r>0.5) with rs564398 [15].

The association of minor risk allele of *SLC2A2* rs11920090 with CVD found in the present study has, to our knowledge, not been shown before. This genetic variant has previously been shown associated with fasting glucose (FG) [5], [16] and is as such considered a type 2 diabetes related variant; interestingly, a genome-wide association study reported that the risk allele of *SLC2A2* rs5400 which is in perfect linkage disequilibrium (LD – r^2^ = 1.0) with the lead SNP of *SLC2A2* rs11920090 [16] was associated with total fasting serum cholesterol level[17] suggesting a possible biological explanation for the statistical relationship between the gene variant and increased risk of CVD. Yet, the association persisted after adjusting for baseline level of serum cholesterol. Since the SNP has been shown to be associated with FG we further did sub analysis in the Inter99 population where baseline FG was available to see if it had any potential mediating effect. The *SLC2A2* variant was significantly associated with baseline FG but adjusting for FG did not change the association between the genetic variant and incident CVD.

The difference in the results between our two examined study samples may be explained by type 1 errors leading to spurious findings that can not be replicated between the two study populations. Another possible explanation is the difference in follow-up time between the two populations. Where MONICA 1 has a mean follow-up time of 23 years the population of Inter99 has only been followed for a mean of ten years. It is likely that with an extended period of follow-up the quality of the baseline measures used for adjustment will change over time since we have to assume the baseline measures are constant. In order to account for possible confounding caused by differences between the two populations the pooled analyses were performed with and without adjustment for cohort without any considerable difference.

Some limitations of the present study need to be addressed. Firstly; the information on diabetes status at baseline is most likely causing residual confounding due to the crude measure. In MONICA 1 self-reported medical doctor-diagnosed diabetes was used. It was not possible to separate type 1 from type 2 diabetes and furthermore diabetes is heavily under diagnosed as previously reported in Inter99[18]. Prevalent diabetes was in Inter99 diagnosed with an OGTT combined with self reported diabetes. Furthermore the analysis would have benefitted from the possibility of including incident diabetes as well but that information was not available. Secondly;.Inter99 was designed as an intervention study [12] and it is possible that the intervention affected the incidence of CVD in the population. However, a recently published Cochrane review shows no effect of individualised intervention on risk of CVD[19] and it is unlikely that it has affected our results. It is possible that limited statistical power causes some of the “null findings” of the majority of the examined genetic variants. On the other hand this study includes 2383 events in more than 120,000 person years.

Several strengths of the study are emphasized. Firstly, the fact that the two populations were recruited from the same geographical area, the baseline examinations were carried out at the same institution and similar phenotyping approaches were used greatly facilitated data comparisons. Secondly; both studies were designed to be used in cardiovascular research and the use of validated endpoints from national registries ensured comparable data of high quality.

## Conclusions

By examining two Danish cohorts we showed that out of the 46 genetic variants examined only the minor risk allele of *SLC2A2* rs11920090 was significantly (*P* = 0.0004) associated with a composite endpoint of incident CVD below the threshold for statistical significance corrected for multiple testing. This association was independent of diabetes status at baseline. Future research should focus on exploring possible pathways associated with the *SLC2A2* variant in linking fasting glucose, type 2 diabetes and ischemic vascular damage.
