# Correction: Genetic Variant *SCL2A2* Is Associated with Risk of Cardiovascular Disease – Assessing the Individual and Cumulative Effect of 46 Type 2 Diabetes Related Genetic Variants

**DOI:** 10.1371/annotation/77e1d9b0-2b94-4a1b-95d5-4336258cecef

**Published:** 2013-05-15

**Authors:** Anders Borglykke, Niels Grarup, Thomas Sparsø, Allan Linneberg, Mogens Fenger, Jørgen Jeppesen, Torben Hansen, Oluf Pedersen, Torben Jørgensen

There was an error in the title of the article. *SCL2A2* should be *SLC2A2*. The full, correct title is:

Genetic Variant *SLC2A2* Is Associated with Risk of Cardiovascular Disease – Assessing the Individual and Cumulative Effect of 46 Type 2 Diabetes Related Genetic Variants

The correct citation is:

Borglykke A, Grarup N, Sparsø T, Linneberg A, Fenger M, et al. (2012) Genetic Variant *SLC2A2* Is Associated with Risk of Cardiovascular Disease – Assessing the Individual and Cumulative Effect of 46 Type 2 Diabetes Related Genetic Variants. PLoS ONE 7(11): e50418. doi:10.1371/journal.pone.0050418 

